# Patient Acceptability of the Use of Advanced Practice Providers in an Outpatient Neurosurgery Clinic

**DOI:** 10.7759/cureus.9157

**Published:** 2020-07-12

**Authors:** Cletus Cheyuo, Nicholas Brandmeir, Natalie Fisher-Perez, Patricia Dekeseredy, Cara Sedney

**Affiliations:** 1 Neurosurgery, West Virginia University, Morgantown, USA; 2 Neurosurgery, Mon Health Systems, Morgantown, USA

**Keywords:** app, midlevel, neurosurgery, nurse practitioner, np, pa, patient satisfaction, physician assistant

## Abstract

Introduction

Increasing demands for healthcare manpower has necessitated the utilization of advanced practice providers (APPs). The effect of APPs in primary care has been well-characterized but is less studied in surgical subspecialties. The objective of this study is to assess the patient acceptability of APPs in an outpatient neurosurgery setting.

Methods

We conducted a prospective, survey-based study among 78 adult patients in the neurosurgical outpatient clinic. The survey consisted of 10 questions assessing the hypothetical acceptability of care provided by neurosurgeons and APPs. These were compared as pre-specified dyads, with patients blinded to dyad composition. The data were analyzed with Chi-square tests.

Results

Patients preferred to see their neurosurgeon for their first clinic visit even with a longer lag time (29% acceptability difference, p = 0.012). Patients also preferred to see the neurosurgeon for their first postoperative visit (20% difference, p = 0.009). For all visits, patients preferred to see an APP if the clinic visit would be on time, rather than see the surgeon with a significant delay (30% difference, p = 0.0002). If their visit was scheduled with an APP, patients preferred that the neurosurgeon review their treatment plan before they left the clinic (15% difference, p = 0.04). Overall, seeing an APP was acceptable if patients were informed ahead of time (37% difference, p < 0.0001).

Conclusions

Team-based care utilizing APPs is acceptable to patients. Patients had strong preferences for seeing their surgeon for the first neurosurgical clinic visit and first post-operative visit. Patients were satisfied with seeing an APP if they could be seen more expeditiously. Patients also preferred to know ahead of time if they were going to see an APP.

## Introduction

Millions of patients are affected by neurosurgical diseases every year, and the cost of neurological diseases overall is estimated at 800 billion US dollars annually [1]. This burden of neurological diseases is not matched by the manpower required for the optimal care of these patients. The global neurosurgeon density is one surgeon per 230,000 people, while the neurosurgeon density in the United States is estimated at one surgeon per 102,775 people [2]. Neurosurgical coverage may further vary by subspecialty [3,4].

In addition to increased numbers of patients requiring healthcare, patient satisfaction is becoming an increasingly important metric in a variety of quality metrics and pay scales [5]. Patient satisfaction is influenced by many factors, including the type of provider, patient wait times and continuity of care. Improved patient satisfaction may correlate positively with better clinical outcomes [6,7]. Advanced practice providers (physician assistants and nurse practitioners; APPs) have become an increasingly important component of the healthcare manpower across all specialties of medicine [8]. Moreover, APPs deliver patient care at all settings of healthcare, including inpatient units, critical care areas, surgical operating rooms, and in outpatient clinics [9-12].

The integration of APPs into the healthcare workforce has significantly supplemented the manpower needs in many medical specialties [9,10,13,14]. The traditional role of APPs is considered to be members of a care team led by a physician (team-based care). Even though APPs have become an integral part of many neurosurgical departments, there is a paucity of literature on the acceptability of utilizing APPs in an outpatient neurosurgical setting from the patient’s perspective. The goal of this research was to assess patient acceptability with regard to the role of APPs in an outpatient, adult neurosurgery clinic. Acceptability is defined as “a multi-faceted construct that reflects the extent to which people delivering or receiving a healthcare intervention consider it to be appropriate, based on anticipated or experienced cognitive and emotional responses to the intervention” [15]. Acceptability comprises seven component constructs including attitude, burden, perceived effectiveness, ethicality, intervention coherence, opportunity costs, and self-efficacy [15].

## Materials and methods

Methods

This is a prospective, survey-based study in which an acceptability questionnaire regarding team-based care was administered to all adult patients presenting to the neurosurgical outpatient clinic regardless of diagnosis or reason for the visit.

Instrument

The acceptability questionnaire was developed by one of the authors of this project with interdisciplinary input from the other members of the research group and clinic. Self-reported measures of acceptability have been previously described [15]. The acceptability theoretical framework utilized for the development of the questionnaire was that of Sekhon and colleagues, with the specific domain under investigation being “affective attitude” towards the implementation process (participant responses to and interactions with the intervention), which has been previously described in the evaluation of the acceptability of complex health interventions [15]. These questions were established through preliminary interviews conducted with patients and aimed to assess the acceptability of team-based care involving APPs within our clinic. This study and the questionnaire were approved by the University’s Institutional Review Board protocol #1812382830.

Procedure

Shortly after the patient was placed in the exam room the patients were met by the research nurse (author PD) and written informed consent was obtained for all participants. The participants were left to complete the survey on their own and the completed survey was collected at the end of their visit. The acceptability survey was constructed using alternative hypothetical dyads which were compared to each other with respect to acceptability on a five-point Likert scale. Power analysis indicated a need for 78 participants to detect a 20% difference in acceptability (with “acceptable” being defined as 4/5 or 5/5 Likert scale rating).

**Figure 1:**
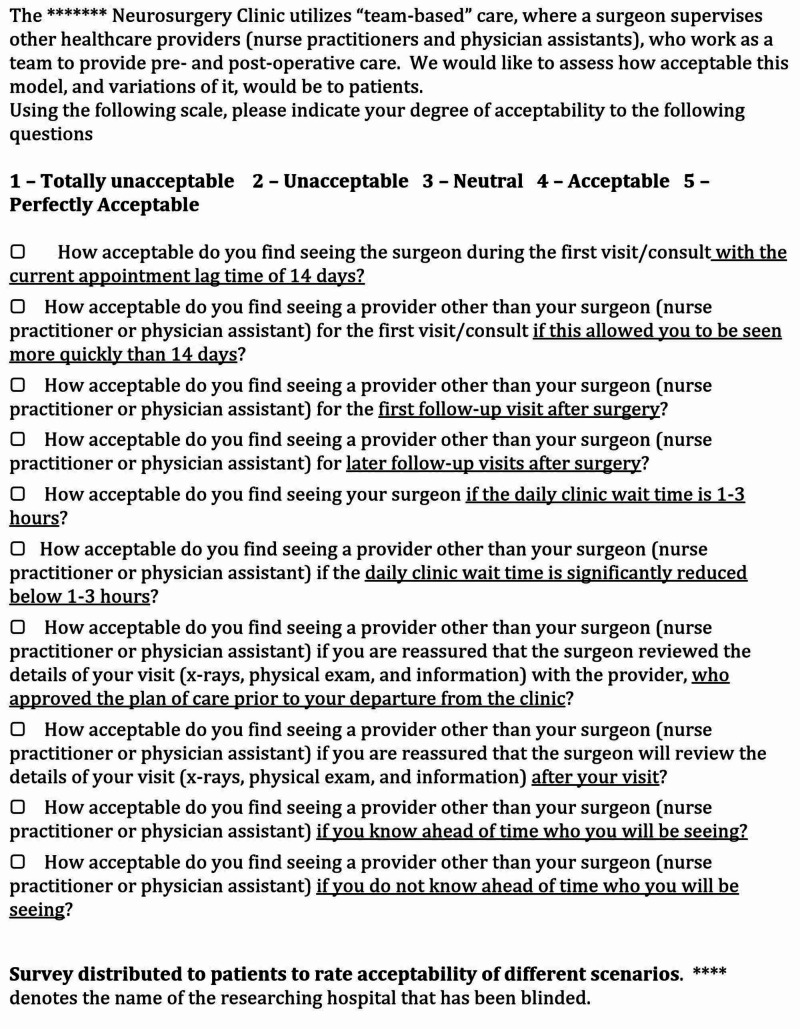
Patient questionnaire

The questionnaire administered to the patients is shown in Figure 1. The survey research was carried out according to best practices as described previously [16].

Statistical analysis

The data were analyzed using Excel (Microsoft, Redmond, WA). Ordinal Likert data were stratified into “acceptable” (>3/5) and “unacceptable" (≤3/5). This division was chosen a priori to the statistical analysis and research activities to allow a more accurate representation of patient preferences and decision making. Because our scale is novel, previous data on its dichotomization does not exist. Rates of acceptability were compared using a 2x2 contingency table and Chi-squared test for significance. Differences were considered significant if p < 0.05 using a modified Bonferroni’s correction to account for multiple comparisons (five comparisons). 

## Results

A total of 78 adult patients (aged 18 years and above), of all genders, responded to the questionnaire over a time period of six weeks. The average age of the clinic population was 54 and the patients ranged between 18 and 93 years of age. Pre-planned analysis at this enrollment demonstrated statistically significant differences and the study was terminated in accordance with our prospective statistical analysis plan. Patients preferred to see their neurosurgeon for their first clinic visit even with a longer lag time (29% acceptability difference, p = 0.012). Patients also preferred to see the neurosurgeon for their first postoperative visit (20% difference, p = 0.009). For all visits, patients preferred to see an APP if the clinic visit would be on time, rather than see the surgeon with a significant delay (30% difference, p = 0.0002). If seeing an APP, patients preferred that the neurosurgeon review their treatment plan before they left the clinic (15% difference, p = 0.04). Overall, seeing an APP was acceptable if patients were informed ahead of time (37% difference, p < 0.0001). Only two outcomes showed acceptability below 50%, waiting >1 hour to see the surgeon and seeing an APP with no advanced warning. The primary results are summarized in Table 1.

 

**Table 1:** Results of survey of patient acceptability of different scenarios Percentage of patients rating a given scenario as acceptable is shown. APP, advanced practice provider. *Statistically significant

	Outcome	‘acceptable’ %	Relative Risk	95% confidence interval	P
Comparison 1: Acceptability of seeing an APP sooner vs. waiting to see the surgeon at initial visit					
	Surgeon with delay	87	1.23	1.04-1.46	0.012*
	APP without delay	70			
Comparison 2: Acceptability of seeing APP at 1^st^ post-op visit vs. APP at subsequent visits					
	APP at 1^st^ visit	51	.71	.55-.92	0.009*
	APP at later visit	72			
Comparison 3: Acceptability of seeing surgeon with >1 hour wait vs. seeing an APP with minimal wait					
	Surgeon with wait	38	.57	.41-.78	0.0002*
	APP without wait	68			
Comparison 4: Acceptability of seeing APP if surgeon reviews case before departing clinic vs. reviews case within a few days					
	Before departure	76	1.25	1.01-1.56	0.04*
	Within 2 days	60			
Comparison 5: Acceptability of seeing APP if informed prior to visit vs. seeing APP with no advanced notice					
	Advanced notice	72	2.07	1.48-2.9	<0.0001
	No advanced notice	35			

## Discussion

Patient interactions with members of the health care team are multifaceted and strongly linked to patient satisfaction [17,18]. In addition, if a patient feels their care met or exceeds expectations, they will judge the quality of that care to be very good. The acceptability of providers is one factor that influences the patient experience, especially how it informs perceptions and expectations of care and thus has the potential to significantly impact patient satisfaction. Furthermore, patient satisfaction has become an important metric for the reimbursement of the healthcare workforce [5]. With an ageing population, there is an increased demand for expansion of the healthcare manpower [5,8]. Healthcare cost for neurological diseases continues to increase, with a current annual cost at 800 billion US dollars [1]. The utilization of APPs in healthcare has proven to be highly acceptable to patients and cost-effective in many surgical and medical specialties [8,10,19].

Even though many neurosurgical services utilize APPs, there is very little literature on the acceptability of this practice. James et al described their pediatric neurosurgical service’s experience with two nurse practitioners and a physician assistant. These APPs were found to be very efficient at providing inpatient and outpatient care, and were also academically very productive [13]. Holleman et al also assessed the satisfaction of neurosurgical staff with the addition of a pediatric neurosurgical nurse practitioner to mitigate resident work-hour restrictions. There was staff satisfaction with the help of the nurse practitioner in running the service [14]. To our knowledge, there has not been a study assessing the patient-rated acceptability of APPs in neurosurgery as part of team-based outpatient care.

We found that for the first visit to the neurosurgical clinic, as well as for the first postoperative visits, patients preferred to see their neurosurgeon rather than the APP. This may relate to the amount of counselling done by the surgeon during the first visit with regard to diagnosis or treatment plan, as well as the ability of the surgeon to answer questions/debrief about the surgery itself during the post-operative visit. However, for other visits (further post-operative care, follow-ups), patients preferred seeing an APP at the clinic if this resulted in less delay to be seen. This finding may suggest that patients may prioritize shorter wait time over continuity of care for follow-up visits other than the initial new patient visit and first post-operative visit. We also found that, in instances where the APP saw the patient, patients preferred to have their treatment plans discussed with the neurosurgeon before leaving the clinic rather than at a later time or not at all, which seems to argue for “parallel” clinics in which the physician is physically present but may not see every patient. In addition, we found that patients strongly preferred to know ahead of time if they were going to see a mid-level provider at the clinic.

Importantly, this study specifically assessed the role of APPs in a team-based care model and therefore such data cannot be extrapolated to have any bearing on the debate regarding APPs as independent care providers.

Limitations

This study is limited by its single-institution nature, and may not be generalizable to other patient populations. A wider geographic sample may be useful for more generalizable results; conversely, this study would be reproducible in local clinic settings for a specific assessment of acceptability in particular patient populations. The assessment of acceptability was limited to the outpatient setting and may not be applicable to other settings such as inpatient or in the operating room. Considerations for future research include accounting for previous experience with APPs, and individual patient’s point in the care trajectory would contribute to a more robust study. The internally developed instrument to assess acceptability was furthermore not assessed for validity or reliability; however, the methods undertaken in this work parallel other attempts at assessing patient self-report of the affective attitude domain of acceptability of complex health interventions.

As the needs of patients increase and surgeon availability becomes more limited, APP utilization is one strategy to meet patient demand for services. Our hope is that the information reported here serves as a starting point for the efficient use of outpatient APPs in a neurosurgical service that meets the needs of both neurosurgeons as well as patients.

## Conclusions

Results from this study support the acceptability of a team-based model of care whereby neurosurgeons work with midlevel providers to deliver care in an outpatient setting. Our study suggests that patients prefer to see their surgeons for the first post-op visit, but in other visits would prefer an APP if able to be seen more expeditiously. Acceptability is greater for parallel clinics in which the plan can be reviewed by the APP and neurosurgeon before the patient leaves, and for advance knowledge of who the patient will be seeing that day.
